# A survival analysis of T1 stage breast cancer and nomogram development: based on the SEER database

**DOI:** 10.1016/j.clinsp.2025.100837

**Published:** 2025-11-23

**Authors:** Lizhi Teng, Yuhan Dong, Juntong Du, Peng Xu, Weiyang Tao

**Affiliations:** a Department of Breast Surgery, The First Affiliated Hospital of Harbin Medical University, Harbin, China; b Key Laboratory of Acoustic, Optical and Electromagnetic Diagnosis and Treatment of Cardiovascular Diseases, Heilongjiang, Harbin, China; c Key Laboratory of Hepatosplenic Surgery, Ministry of Education, Harbin, Heilongjiang, China; d NHC Key Laboratory of Cell Transplantation, Heilongjiang, China

**Keywords:** Breast cancer, T1a, T1b and T1c, Nomogram, Survival, Treatment

## Abstract

**Introduction:**

Breast Cancer (BC) is a widespread non-cutaneous malignancy. T1 stage of small tumors are generally considered to have a favorable prognosis, but controversy persists regarding their treatment and prognosis.

**Methods:**

The cohort included clinicopathological characteristics of BC patients along with treatment information from T1 in the Surveillance, Epidemiology and End Results program (SEER) database from 2010‒2015. Construct a nomogram based on the variables identified by the multifactorial analysis results.

**Results:**

A total of 164,906 female patient records were obtained for enrolment. Patients in the training set of T1 had a 3-, 5- and 10-years of OS for 95.2 %, 90.9 % and 84.4 % as well as a 3-, 5- and 10-years of BCSS for 98.4 %, 97.0 % and 95.4 %. Multivariate analysis found age, race, grade, T-stage, N-stage, chemotherapy, radiation, surgery, Estrogen Receptor (ER) status, Progesterone Receptor (PR) status and Human Epidermal growth factor Receptor-2 (HER2) status to be associated with OS. And age, grade, T-stage, N-stage, chemotherapy, radiation, surgery, diagnosis to treatment, ER-status, PR-status, and HER2-status are associated with BCSS. Independent prognostic risk factors incorporated into the construction of a nomogram. The AUC values for 3-, 5- and 10-years of OS and BCSS were greater than 0.7. The ROC, calibration and DCA curves verified that the nomogram had better predictability and benefits.

**Conclusion:**

The outcomes demonstrated a disparity in prognosis between groups T1a, T1b, and T1c. The constructed nomogram is able to predict OS and BCSS for 3-, 5- and 10-year periods reasonably well.

## Highlights

• The prognosis for small tumors in stage T1 breast cancer is not always favorable.
• Identify independent prognostic risk factors for the T1 stage.
• •Using nomograms to assist clinical treatment and predict prognosis in the T1 stage.

## Introduction

Breast Cancer (BC) is the most common non-cutaneous malignant tumor and is the leading cause of cancer deaths in women worldwide.^1^ According to the newly released GLOBOCAN report, BC (11.7 %) has surpassed lung cancer (11.4 %), with an estimated 2.3 million cases on 2020, and the incidence rate is on the rise.^2^^,^^3^ Given the high prevalence of BC and the enormous threat it poses to women's health, it has risen to be a major public health issue that cannot be avoided. Over the past century, early diagnosis of BC has been made possible by the use of mammography programs and the spread of BC screening programs in several countries.^4^^,^^5^ It also leads to considerably better detection of carcinoma in situ and small invasive tumors, especially for T1a-b, which accounted for 23 % of invasive BC < 1 cm diagnosed in 2010.^6^^,^^7^

The updated American Joint Committee on Cancer's (AJCC) Staging System classified BC based on tumor size. The tumor with a diameter of ≤ 2 cm is described as T1. Within the T1 subgroup, the subgroup of tumors less than or equal to 0.5 cm is called T1a; tumors >0.5 cm and less than or equal to 1 cm are referred to as T1b; and tumors between 1 cm and 2 cm are defined as T1c.^8^

Detection of small tumors is becoming more common, and experts have also shown that this type has a better prognosis and can be exempted from systemic adjuvant therapy.^9^ According to most cancer management guidelines, including ones from the National Comprehensive Cancer Network (NCCN) and the St. Gallen International Consensus Guidelines, tumors classified as T1 are generally considered to have a better prognosis. Adjuvant systemic chemotherapy is only recommended for patients with Triple-Negative Breast Cancer (TNBC) and HER2-positive tumors. Treatment decisions for patients with T1a stage tumors are based on individual patient characteristics and clinical experience.^10^^,^^11^ Consensus statements indicate that T1-stage tumors can be treated with downgraded therapy, but the reality is far from that. This small tumor is a “wolf in sheep's clothing”.

The AJCC identified primary tumor size, regional lymph node status, and distant metastasis as the three key prognostic factors.^12^ The biological characteristics of tumors suggest that small tumors can occasionally be more aggressive and that several tumors may have already metastasized before clinical detection.^12^, ^13^ Currently, published treatment guidelines do not explicitly address small tumors, particularly for patients with T1aN0M0 and T1bN0M0 tumors. Because prognostic risk factors for the T1 stage are rarely discussed, clinicians may underestimate the malignancy of these tumors when making treatment decisions. Therefore, the focus of current research has become clarifying the prognosis of T1a, T1b, and T1c breast cancer tumors, as well as assessing clinical and pathological features closely related to the prognosis of T1-stage tumors.

The authors accessed the SEER database to obtain the clinical and pathological information, as well as the survival data, of patients in the T1 group from 2010 to 2015. The authors identified independent risk factors for poor prognosis in this group and created a nomogram to quantify these factors and guide clinical treatment decisions.

## Methods

### Data source

The data were abstracted from 18 registry in the Surveillance, Epidemiology, and End Results Program (SEER) database (http://seer.cancer.gov/), which is one of the largest publicly available cancer datasets, covering approximately 48 % of the U.S. population. The database mainly includes clinicopathological characteristics data, treatment details and survival of the patients. Following the strict application and registration conditions for the SEER database, permission to access the database was eventually granted with an exemption from the right to informed consent. The study design strictly followed the STARD guidelines (Supplement 4).

For patients diagnosed with female breast cancer in T1a/b/c stage between 2010‒2015, which was collected by applying SEER*Stat 8.4.0 software with 195,909 patient records screening. Demographic data consisted of clinicopathological characteristics, mainly age, race (White, Black or Other), grade (I, II, III or IV), pathology (Mainly including infiltrating duct carcinoma), T-stage (T1a, T1b or T1c), N-stage (N0, isolated tumor cells: ITC, micrometastases: Mic, N1, N2 or N3) and diagnosis to treatment time, while treatment information, mainly chemotherapy, radiotherapy and surgery (Unknow, Axillary lymph node dissection: ALND or Non-axillary lymph node dissection: Non-ALND), as well as the patient's long-term survival status. Enrolment criteria for the current study include: 1) Female patient; 2) Single tumor in breast cancer; 3) Age≥ 18-years; 4) Single breast cancer; 5) T1 stage. And exclusion criteria include: 1) Male; 2) Patients with more than one primary tumor; 3) Age < 18; 4) Neoadjuvant chemotherapy or targeted therapy; 5) Existence of distant metastases; 6) Patients with incomplete clinicopathological characteristics, treatment information or survival information. Adhering to the above criteria, a total of 164,906 patients were eventually included for the purpose of survival and prognostic analyses as well as nomogram construction. Survival outcomes were categorized as Overall Survival (OS), which was defined as the disease of the patient from any cause up to the time and Breast Cancer-Specific Survival (BCSS), which was defined as the disease of the patient from breast cancer up to the time based on survival status during follow-up.

### Statistical analysis and nomogram construction

To ensure a balance between maximizing training data and minimizing validation set evaluation variance, as well as reliable evaluation, the authors applied the clustering method used for large sample datasets in the past. Ultimately, the authors chose a 7:3 ratio for dividing the training and validation sets.^14^^,^^15^ The acquired data (*n* = 164,906) were randomly divided into two subgroups, the training set and the validation set. The Chi-Square test was employed to detect the differences among the two groups to ensure that they were randomized (*p* ≥ 0.05). In the training, clinical baseline information and treatment information were univariate analyzed with the log-rank test, and statistically significant variables (*p* < 0.05) were screened for development of multivariate COX hazard regression models. Hazard Ratios (HRs) and 95 % Confidence Intervals (95 % CIs) were used. In multivariate analyses, the statistically significant variables were identified as independent prognostic risk factors and Kaplan-Meier curves were constructed for survival comparisons between groups or variables. The above operations were achieved by applications of SPSS 22.0.

In the training set, independent prognostic risk factors were cohorted for nomogram construction (packages: rms), which aim to predict OS and DFS at 3-, 5- and 10-years. The authors performed several evaluations with the validation set to validate the performance of the nomogram in clinical prediction. The accuracy of nomogram prediction was assessed by plotting Receiver Operating Characteristic (ROC) curves (packages: risk Regression and survival) and calculating Area Under Curve (AUC) values, which AUC > 0.7 proved well performed in the nomogram. Calibration curves (packages: rms and survival) confirm the consistency between actual and predicted incidence in the clinical setting, which uses the bootstrap method with 100 replications. Decision Curve Analysis (DCA) curve (packages: ggDCA, rms, and survival) was graphed to verify the clinical practicability of the nomogram and the assessment of survival benefit.

## Result

### Clinicopathological characteristics of T1a, T1b, and T1c patients

A total of 195,909 female patients with clinicopathological records from the SEER database during 2010‒2015, following screening for inclusion and exclusion criteria, the final available information was 164,906 patients. These patients were randomized to the training set (*n* = 115,434) and validation set (*n* = 49,472) according to a ratio of 7 to 3, and clinicopathological characteristics is shown in Table 1. The training set was used for survival analyses as well as for the construction of nomograms, and the validation set was used for the validation of the clinical performance of nomograms.

**Table 1:** Demographic distribution of the training set and validation set of T1a\b\c breast cancer patients.

	Total number ( %)	Training sets ( %)	Validation sets ( %)	p
**Total**	164 906 (100.0)	115 434 (70.0)	49 472 (30.0)	
**Age**				
< 50	27 562 (16.7)	19 233 (16.7)	8 329 (16.8)	
≥50	137 344 (83.3)	96 201 (83.3)	41 143 (83.2)	0.358
**Race**				
White	135 692 (82.3)	94 963 (82.3)	40 729 (82.3)	
Black/Other	29 214 (17.7)	20 471 (17.7)	8 743 (17.7)	0.765
**Grade**				
G1	54 635 (33.1)	38 190 (33.1)	16 445 (33.2)	
G2	75 721 (45.9)	53 182 (46.1)	22 539 (45.6)	
G3\G4	34 550 (21.0)	24 062 (20.8)	10 488 (21.2)	0.115
**Pathology**				
Infiltrating duct carcinoma	126 904 (77.0)	88 948 (77.0)	37 956 (76.7)	
Lobular carcinoma	12 737 (7.7)	8 881 (7.7)	3 856 (7.8)	
Other	25 265 (15.3)	17 605 (15.3)	7 660 (15.5)	0.337
**T-stage**				
T1a	19 753 (12.0)	13 867 (12.0)	5 886 (11.9)	
T1b	49 674 (30.1)	34 773 (30.1)	14 901 (30.1)	
T1c	95 479 (57.9)	66 794 (57.9)	28 685 (58.0)	0.791
**N-stage**				
N0	134 472 (81.5)	94 155 (81.6)	40 317 (81.4)	
ITC/Mic	9 697 (5.9)	6 751 (5.8)	2 946 (6.0)	
N1/N2/N3	20 737 (12.6)	14 528 (12.6)	6 209(12.6)	0.696
**Chemotherapy**				
No	121 233 (73.5)	84 956 (73.6)	36 277 (73.3)	
Yes	43 673 (26.5)	30 478 (26.4)	13 195 (26.7)	0.257
**Radiation**				
No	75 614 (45.9)	52 810 (45.7)	22 804 (46.1)	
Yes	89 292 (54.1)	62 624 (54.3)	26 668 (53.9)	0.197
**Surgery**				
No	3 399 (2.1)	2 396 (2.1)	1 003 (2.0)	
Non-ALND	148 210 (89.8)	103 712(89.8)	44 498 (90.0)	
ALND	13 297 (8.1)	9 326 (8.1)	3 971 (8.0)	0.761
**Diagnosis to treatment (months)**				
≤ 1	121 757 (73.8)	85 216 (73.8)	36 541 (73.9)	
> 1	43 149 (26.2)	30 218 (26.2)	12 931 (26.1)	0.866
**ER-status**				
Negative	19 248 (11.7)	13 406 (11.6)	5 842 (11.8)	
Positive	145 658 (88.3)	102 028 (88.4)	43 630 (88.2)	0.258
**PR-status**				
Negative	35 008 (21.2)	24 458 (21.2)	10 550 (21.3)	
Positive	129 898 (78.8)	90 976 (78.8)	38 922 (78.7)	0.532
**HER2-status**				
Negative	146 693 (89.0)	102 721 (89.0)	43 972 (88.9)	
Positive	18 213 (11.0)	12 713 (11.0)	5 500 (11.1)	0.536

The overwhelming majority of T1a, T1b, and T1c female breast cancer patients are aged ≥ 50-years and are disproportionately white. While the pathology was invasive carcinoma accounted for 77.0 % of cases and the tumor grade tended to be lower for G1 (33.1 %) and G2 (46.1 %). Among the T1 stage, more than half of the cases were T1c and most of the patients presented non-axillary lymph nodes. With Non-ALND being the predominant strategy of selection, and a minority of patients opting for postoperative adjuvant chemotherapy (26.4 %), while more options for radiotherapy (54.3 %). In the various breast cancer subtypes, Hormone Receptor positive (HR+) was 88.4 % while Human Epidermal growth factor Receptor-2 positive (HER2+) was only 11 %.

### Survival and prognosis analysis

**Fig. 1:**
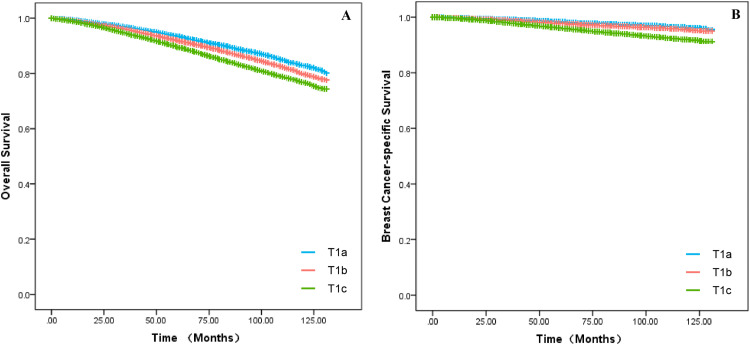
**The survival outcomes for OS and BCSS in the T1a, T1b and T1c groups**. Kaplan-Meier curves were applied to compare survival between T1a, T1b and T1c groups. (A) The status of OS in the subgroups of T1. (B) The status of BCSS in the subgroups of T1.

In the training set, baseline data were collected for all breast cancer patients with T1a, T1b and T1c between 2010 and 2015, which were analyzed for all clinicopathological characteristics along with survival information. Patients in the training set had a 3-, 5- and 10-years of OS for 95.2 %, 90.9 % and 84.4 % as well as a 3-, 5- and 10-years of BCSS for 98.4 %, 97.0 % and 95.4 %. The difference in survival between T1a, T1b and T1c in OS was progressively more apparent with time; however, long-term survival was similar between T1a and T1b in BCSS, which both had a better prognosis than T1c (Fig. 1A‒B). Independent prognostic risk factors were identified by using univariate log rank analysis (Table 2), which included multivariate analysis for statistically significant (*p* < 0.05) and multivariate COX hazard regression models (Table 3).

**Table 2:** Univariate analysis conducted for T1a\b\c breast cancer patients.

	OS			BCSS		
	Mean OS (Estimated value), mo	95 % CI	p (Log rank)	Mean BCSS (Estimated value), mo	95 % CI	p (Log rank)
Age						
< 50	125	125.638‒126.233		127	126.900–127.422	
≥ 50	115	114.957–115.393	<0.001	126	126.277–126.539	<0.001
**Race**						
White	116	116.515–116.935		126	126.560–126.815	
Black	113	113.150–114.596		123	123.299–124.326	
Other	121	121.411–122.441	<0.001	127	127.280–127.942	<0.001
**Grade**						
G1	118	118.057–118.683		128	128.476–128.776	
G2	116	116.701–117.258		126	126.664–126.997	
G3\G4	114	114.265–115.159	<0.001	122	122.260–122.953	<0.001
**Pathology**						
Infiltrating duct carcinoma	117	116.930–117.361		126	126.305–126.575	
Lobular carcinoma	116	115.832–117.210		126	125.831–126.699	
Other	116	115.826–116.809	0.001	127	126.896–127.455	<0.001
**T-stage**						
T1a	120	120.049–121.003		128	128.299–128.800	
T1b	118	118.084–118.742		127	127.684–128.047	
T1c	115	115.218–115.739	<0.001	125	125.245–125.588	<0.001
**N-stage**						
N0	117	117.100–117.516		127	127.259–127.495	
ITC	119	117.887–120.308		127	126.542–127.986	
Mic	118	117.140–118.996		125	125.352–126.593	
N1	116	115.702–116.914		123	122.992–123.925	
N2	107	105.660–109.239		115	113.773–116.961	
N3	95	92.115–98.815	<0.001	100	97.284–103.864	<0.001
**Chemotherapy**						
No	115	115.398–115.856		127	127.141–127.394	
Yes	120	120.305–120.957	<0.001	124	124.321–124.855	<0.001
**Radiation**						
No	112	111.727–112.368		125	124.831–125.237	
Yes	121	120.892–121.326	<0.001	127	127.628–127.894	<0.001
**Surgery**						
No	73	71.735–75.962		104	102.767–106.861	
ALND	118	118.064–118.446		127	127.163–127.389	
Non-ALND	113	112.763–114.192	<0.001	123	122.536–123.600	<0.001
**Diagnosis to treatment (months)**						
≤ 1	117	117.229–117.661		126	126.555–126.822	
> 1	115	115.207–115.992	<0.001	126	125.845–126.335	<0.001
**ER-status**						
Negative	112	112.088–113.359		121	120.681–121.695	
Positive	117	117.328–117.723	<0.001	127	127.126–127.354	<0.001
**PR-status**						
Negative	113	113.406–114.311		123	122.763–123.438	
Positive	117	117.600–118.014	<0.001	127	127.345–127.580	<0.001
**HER2-status**						
Negative	116	116.612–117.015		126	126.529–126.775	
Positive	118	117.712–118.827	<0.001	125	125.239–126.012	<0.001

**Table 3:** Univariate and multivariate analysis conducted for T1a\b\c breast cancer patients.

	OS			BCSS		
	p (Log rank)	HR (95 % CI)	p (Cox)	p (Log rank)	HR (95 % CI)	p (Cox)
**Age**	<0.001	3.283 (3.086–3.494)	<0.001	<0.001	1.462 (1.354–1.578)	<0.001
**Race**	<0.001	0.870 (0.848–0.893)	<0.001	<0.001	0.962 (0.921–1.005)	0.080
**Grade**	<0.001	1.174 (1.146–1.202)	<0.001	<0.001	1.555 (1.487–1.626)	<0.001
**Pathology**	0.001	1.013 (0.993–1.033)	0.192	<0.001	0.997 (0.959–1.036)	0.876
**T-stage**	<0.001	1.250 (1.222–1.279)	<0.001	<0.001	1.347 (1.285–1.411)	<0.001
**N-stage**	<0.001	1.205 (1.190–1.221)	<0.001	<0.001	1.412 (1.385–1.440)	<0.001
**Chemotherapy**	<0.001	0.508 (0.486–0.531)	<0.001	<0.001	0.827 (0.773–0.885)	<0.001
**Radiation**	<0.001	0.491 (0.476–0.506)	<0.001	<0.001	0.531 (0.503–0.561)	<0.001
**Surgery**	<0.001	0.577 (0.550–0.605)	<0.001	<0.001	0.590 (0.546–0.637)	<0.001
**Diagnosis to treatment (months)**	<0.001	0.979 (0.947–1.012)	0.214	<0.001	0.930 (0.876–0.988)	0.019
ER-status	<0.001	0.758 (0.715–0.802)	<0.001	<0.001	0.718 (0.656–0.787)	<0.001
**PR-status**	<0.001	0.825 (0.788–0.863)	<0.001	<0.001	0.688 (0.635–0.745)	<0.001
**HER2-status**	<0.001	0.913 (0.866–0.962)	0.001	<0.001	0.787 (0.725–0.855)	<0.001

Univariate analysis detected all the variables to be statistically significant in OS and BCSS (Table 2) in T1a, T1b, and T1c patients’ groups. Therefore, all the selected variables were involved in the multivariate analysis as shown in Table 2, age, race, grade, pathology, T-stage, N-stage, chemotherapy, radiation, surgery, diagnosis to treatment, Estrogen Receptor (ER) status, Progesterone Receptor (PR) status and HER2-status associated with OS. While age, race, grade, pathology, T-stage, N-stage, chemotherapy, radiation, surgery, diagnosis to treatment, ER-status, PR-status and HER2-status are associated with BCSS.

### Construction a novel nomogram for OS and BCSS

**Fig. 2:**
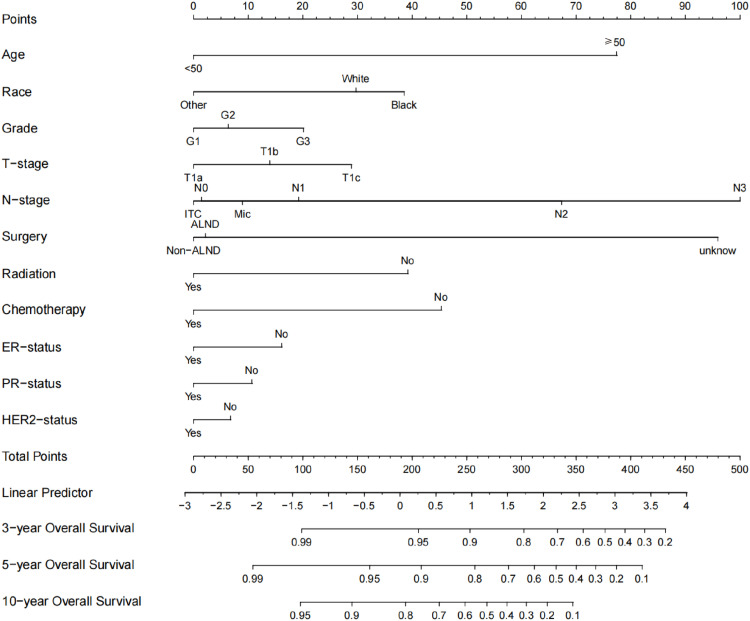
**Construction of a nomogram for 3-year, 5-year and 10-year OS in patients**. Construction of nomograms predicting 3-, 5-, and 10-years OS in patients. According to the patient information where each clinicopathological feature corresponds to a point at the top of the chart, the sum of all variables corresponds to a total point, and the bottom line perpendicular to the total point is the 3-, 5- and 10-years OS.

**Fig. 3:**
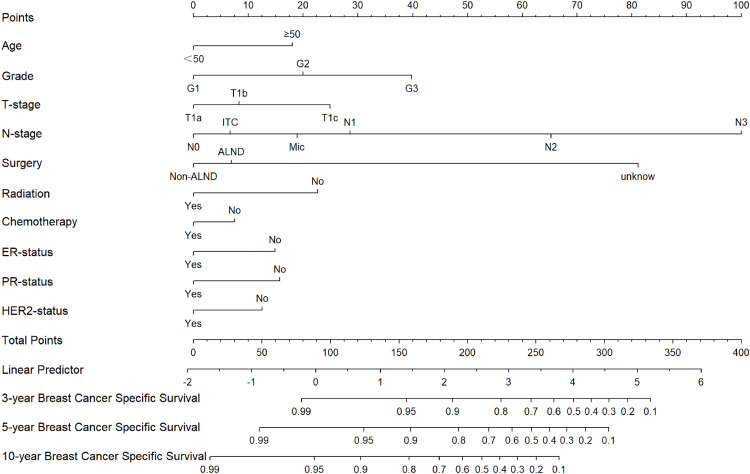
**Construction of a nomogram for 3-year, 5-year and 10-year BCSS in patients**. Construction of nomograms predicting 3-, 5-, and 10-years BCSS in patients. According to the patient information where each clinicopathological feature corresponds to a point at the top of the chart, the sum of all variables corresponds to a total point, and the bottom line perpendicular to the total point is the 3-, 5- and 10-years BCSS.

Determination of independent prognostic risk factors associated with OS and BCSS in T1a, T1b and T1c in multivariate COX hazard regression models, where the variables with statistically significant (*p* < 0.05) were ultimately included for the construction of the nomogram (Fig. 2‒3). Consequently, the nomogram for predicting OS incorporated eleven variables, as well as the variables were statistically significant in the BCSS following the nomogram construction. It was found that diagnosis to treatment accounted for a negligible portion of the overall score for prediction (score < 2), so the nomogram was plotted for only ten variables.

**Fig. 4:**
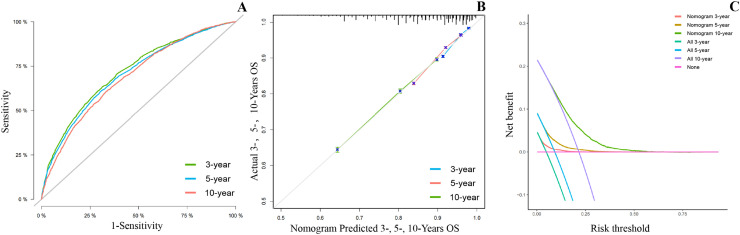
**Validating the advantage of the nomogram in OS**. Verifying the predictive superiority of nomogram in OS.

**Fig. 5:**
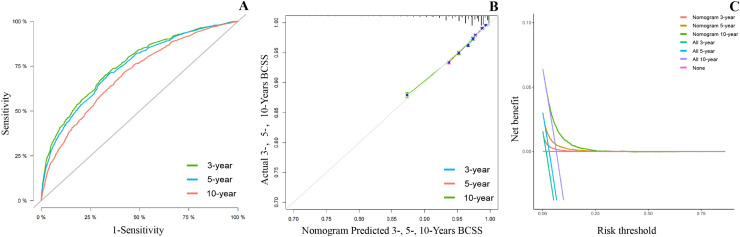
**Validating the advantage of the nomogram in BCSS**. Verifying the predictive superiority of nomogram in BCSS.

Validation of the results demonstrated that both sets of results were sufficiently accurate for predicting OS and BCSS. For the training set, ROC curves were plotted to obtain AUC values, which for 3-, 5- and 10-years of OS (Fig. 4A) were 71.8 (70.5‒73.0), 70.4 (69.4‒71.3) and 68.9 (67.8‒70.1) and for 3-, 5- and 10-years of BCSS (Fig. 5A) were 75.7 (73.8‒77.6), 74.4 (72.9‒75.9) and 70.1 (68.4‒71.8). Meanwhile, in the validation set, for 3-, 5- and 10-years of OS (S1A) were 72.7 (70.9‒74.5), 70.2 (68.8‒71.6) and 67.1 (65.4‒68.9) and for 3-, 5- and 10-years of BCSS (S2A) were 78.1 (75.3‒81.0), 76.6 (74.3‒78.8) and 70.1 (67.4‒72.8). Plotting calibration curves to predict the accuracy of the nomogram for OS (Fig. 4B, S1B) and BCSS (Fig. 5B, S2B) assessment revealed that no significant deviation was found between observed and predicted probabilities, so the nomogram had high predictive accuracy. The DCA curve also suggests that the nomogram has high clinical utility as well as decision-making value to improve the maximum survival benefit for patients for OS (Fig. 4C, S1C) and BCSS (Fig. 5C, S2C).

## Discussion

Breast cancer, as a heterogeneous disease, is on the increase worldwide. As breast cancer screening techniques are constantly improving, the authors are diagnosing an escalating percentage of early-stage breast cancers, especially small tumors.^1^, ^2^, ^3^ The study examined the impact of T1a, T1b, and T1c on OS and BCSS, while identifying independent prognostic risk factors. By constructing a nomogram, the authors quantified the influence of different clinical pathological characteristics on prognosis and identified risk factors associated with a poorer prognosis. Based on the nomogram's quantification, the authors offer guidance for clinical treatment decisions regarding T1 staging.

The management of tumors should not be based on a “binary” concept of tumor size, rather, it should take into account a variety of factors.^16^ According to some studies, specific molecular subtypes at stages T1a and T1b exhibit no correlation between prognosis and age.^17^ However, this appears to contradict the conclusion of Rachel L. Theriault et al., who noted that the recurrence-free survival rate for patients over 50-years of age was 4.98 times higher than for patients aged 35-years or younger (95 % CI 2.91‒8.53), while the distant recurrence-free survival rate in stages T1a and T1b was 4.7 times higher (95 % CI 2.51‒8.79).^18^ For patients over 70-years of age, the risk of death is higher in the T1bN0 stage than in the T1aN0 stage.^19^ It is consistent with our conclusion that age is strongly associated with both OS and BCSS. The proportion of ≥ 50-years of age in OS was considerably greater than in BCSS in the nomogram. The reason for this may be attributed to, firstly, the inevitable occurrence of normal deaths of patients in the older age cohorts and, secondly, the similarly increased incidence of cardiovascular disease and other complications in older patients, leading to deaths caused by other diseases. Unfortunately, the authors were unable to conduct further investigation due to the lack of medical records on comorbidities and other confounding factors in the SEER database.

Previous reviews have undeniably correlated the grade of the tumor with the prognosis in T1a, T1b and T1c.^17^^,^^19^ Compared to G1 patients, those with high-grade tumors are exposed to a greater risk for cancer fatality, and the 10-years recurrence-free survival rate may be <75 %.^20^ The authors also corroborate this conclusion, but small T1a and T1b tumors occasionally seem to have a higher biological aggressiveness. Among the data, axillary lymph node metastases occurred for 12.5 of patients with G1 tumors, 19.7 % of patients with G2 tumors and 25.4 % of patients with G3 tumors. However, whether it could demonstrate a direct relationship between tumor grade and tumor aggressiveness still needs to be further investigated.

Experts have tacitly accepted the prognosis for T1 tumors as having two groups, T1a,b and T1c, based on previous retrospective studies. According to research by Abdilkerim Oyman and Mamta Parikh et al., there is no statistically significant difference in the BCSS between T1a and T1b tumors.^21^^,^^22^ And studies also confirmed that, for specific molecular subtypes, the prognosis for T1c tumors is worse than for T1a and T1b tumors (BCSS: HR = 3.847, *p* < 0.001; OS: HR = 2.055, *p* < 0.001).^23^ Established conclusions are consistent with the results of this work. Firstly, it was found in Fig. 1A that T1a, T1b and T1c were significantly different in OS, however, it seems that T1a and T1b are not significantly different from T1c over 5-years. In Fig. 1B, T1a, b hardly differed in long-term survival, but T1c clearly had a worse prognosis. Secondly, the authors cannot agree that the two can be lumped together for T1a and T1b although they are similar. It can clearly be seen in the nomogram that there is still a definite risk gap between T1a and T1b (Fig. 2‒3), as well as a better long-term survival for T1a (S3A‒B: OS and BCSS; *p* < 0.001). Finally, summarizing the above conclusions, whether the authors make 1 cm a suitable node for BC in future research, it is clear that this is not applicable in OS.

**Fig. 6:**
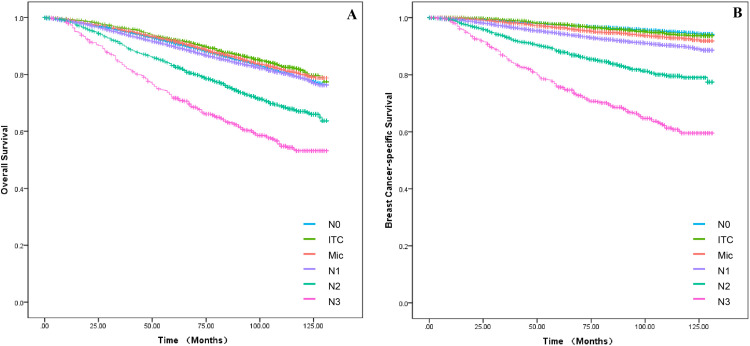
**The OS and BCSS of patients with N stage**. Kaplan-Meier curves were applied to compare survival in N stage. (A) The status of OS in the subgroups of N stage. (B) The status of BCSS in the subgroups of N stage.

Axillary Lymph Node Metastasis (ALNM) is a well-established risk factor for breast cancer. A prospective study of T1 stage found that N0 was more common. However, this was unrelated to the number of positive axillary lymph nodes (N1 vs. N0, HR = 1.25, 95 % CI: 1.17–1.32).^24^ These results imply that some smaller tumors may be more aggressive and require more thorough axillary lymph node dissection for T1 stage tumors. Additionally, there was no statistically significant difference in breast cancer-specific mortality rates between the N0, N1 and N2+ groups in T1 tumors.^12^^,^^20^ Each of the above studies has demonstrated the prognostic correlation between N stage and T1 stage, but they seem to have overlooked the discussion of ITC and Mic. It is also evident from our findings that ALNM is strongly associated with OS and BCSS in T1. Firstly, N0 patients still account for the majority of patients (81.5 %), but it cannot be denied that small tumours still present a high degree of aggressiveness (N1+: 18.5 %) and the decision on treatment needs to be made according to the characteristics of the tumour (pathology and grade et al.) as well as the individual patient (age, N-stage). Secondly, the authors found that in the survival curves of ALNM, it was difficult to observe a meaningful effect of N0, ITC and Mic in OS (Fig. 6A; *p* < 0.001), which were all better than N1, but the results were not clear. These disparities were evident in the BCSS (Fig. 6B; *p* < 0.001), which was not significant in the N0 vs. ITC groups, all being significantly higher than the Mic group. Finally, the data comes from the period 2010‒2015, which inevitably suffers from a number of technical problems, thus resulting in omissions from ITC and Mic.^25^^,^^26^ On the other hand, since there are no guidelines indicating treatment strategies for ITC and Mic norms, overtreatment may occur in clinical care. Therefore, the proportion of ITC and Mic observed in the nomogram of the conclusion seems to account for a much larger proportion, which also requires continuous advances in clinical techniques and pathology to ensure more accurate conclusions.

**Fig. 7:**
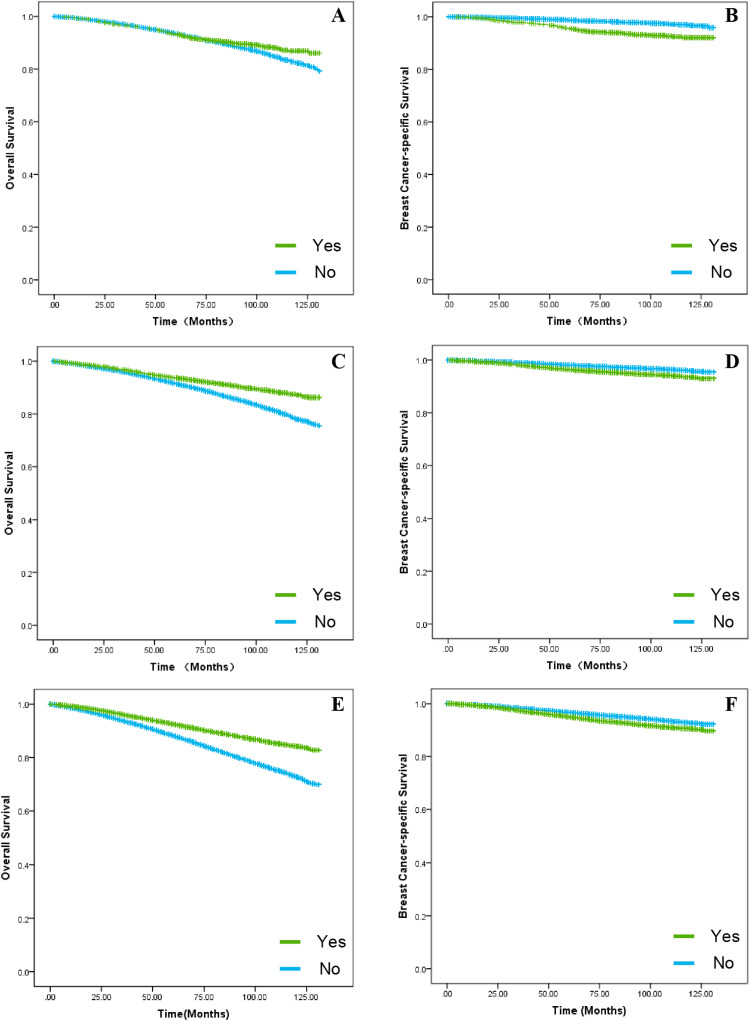
**The effect of chemotherapy on survival rates in the T1a, T1b and T1c groups**. Kaplan-Meier curves were applied to compare the results of chemotherapy on survival in the T1a, T1b and T1c groups. (A) The impact of chemotherapy on OS in patients in the T1a group. (B) The impact of chemotherapy on BCSS in patients in the T1a group. (C) The impact of chemotherapy on OS in patients in the T1b group. (D) The impact of chemotherapy on BCSS in patients in the T1b group. (E) The impact of chemotherapy on OS in patients in the T1c group. (F) The impact of chemotherapy on BCSS in patients in the T1c group.

When it comes to the treatment of tumors, the authors follow the principle of “less is more”. According to research, experts agree that chemotherapy does not significantly improve OS or BCSS in T1a patients.^4^^,^^27^ However, it is believed that chemotherapy significantly improves OS in the T1b and T1c subgroups but does not improve BCSS.^28^^,^^29^ These conclusions were also verified in our findings. Chemotherapy has a survival benefit for T1a, T1b, and T1c as well as the benefit increases gradually over time (Fig. 7A; *p* = 0.033, Fig. 7C, E; *p* < 0.001). Of these, chemotherapy within 5-years of T1a appears to have no significant benefit for OS, which may be a bias in conclusions due to insufficient previous follow-up time (Fig. 7A; *p* = 0.033). However, the conclusions were quite varied in the BCSS, with results showing no survival benefit of chemotherapy for any of the three subgroups (Fig. 7B, D, F; *p* < 0.001). As such, chemotherapy does not seem to be relevant for T1 group BC by itself, but the authors also cannot deny the enhancement for OS. The existence of a discrepancy between OS and BCSS, which are considered in the context of other possible clinical risk factors, such as recurrence locally versus distantly.

The availability of various BC subtypes is essential for the prognosis of the patient and for the options of treatment. Changjun Wang et al. compared various subtypes of BC in their outcome and found that TNBC patients had the worst prognosis in T1a.^30^ G Cancello et al. demonstrated an increased rate of local recurrence (HR = 4.53; 95 % CI: 1.56‒13.1) and distant metastases with decreased BCSS (HR = 3.22; 95 % CI +1.44‒7.18) and OS (HR = 2.87; 95 % CI: 0.05‒7.89) in HER2+ BC patients.^31^ With respect to the diverse BC subtypes, the nomogram indicated that the weighting of the subvariables was similar in OS and BCSS. Moreover, our results similarly suggested that TNBC had the worst prognosis in the T1 group, followed by HER2+. Depending on the prognosis of the different subtypes, individualized treatment becomes more critical.

Although the outcomes incorporate a tremendous amount of clinicopathological information, there are still some shortcomings. Firstly, neoadjuvant chemotherapy data is unavailable, making it impossible to further assess survival and prognosis in patients with small tumors in ALNM. Secondly, due to the lack of information on chemotherapy regimens, comparisons of prognosis under the same adjuvant chemotherapy regimen are not possible. Finally, HR+ and HER2+ are clearly essential prognostic risk factors, but the absence of endocrine therapy and targeted therapy precludes further discussion of the treatment.

## Authors’ contributions

Weiyang Tao: Conception and design; data acquisition and assembly; data analysis and entry; graphic drawing; manuscript writing.

Lizhi Teng: Conception and design; Data acquisition and assembly; Data analysis and entry; Graphic drawing; Manuscript writing.

Juntong Du: Graphic drawing.

Yuhan Dong: Data acquisition and assembly.

Peng Xu: Graphic drawing.

Final approval of manuscript: All authors.

## Ethical approval

Because SEER uses nonidentifiable patient information, institutional review board approval was not required for this research. The right to informed consent was waived because the study was based strictly on the registry.

## Data availability

The datasets generated during and/or analyzed during the current study are available in the SEER database repository, Surveillance, Epidemiology, and End Results Program (cancer.gov).

## Funding

This work was supported by The First Affiliated Hospital of Harbin Medical University Fund for Distinguished Young Medical Scholars (2021J17) and Beijing Medical Award Foundation (YXJL-2021–0302–0287).

## Declaration of competing interest

The authors report no conflict of interest concerning the materials or methods used in this study or the findings specified in this paper.

## Appendix Supplementary materials

Image, application 1
