# Supplementary material for: A survival analysis of T1 stage breast cancer and nomogram development: based on the SEER database
Source: Clinics (Sao Paulo). 2025 Nov 23;80:100837. doi: 10.1016/j.clinsp.2025.100837 (PMC12681841; doi:10.1016/j.clinsp.2025.100837)

**CLINICS-D-25-00017**

**Supplementary Material**

**Figure S1** **Validating the advantage of the nomogram in OS.** Verifying the predictive superiority of nomogram in OS.


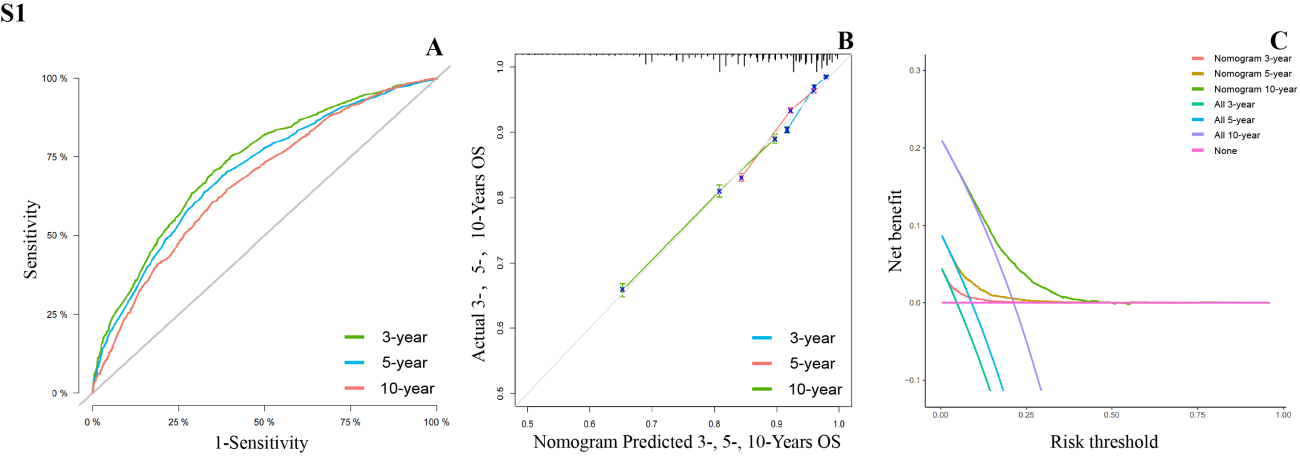


**Figure S2** **Validating the advantage of the nomogram in BCSS.** Verifying the predictive superiority of nomogram in BCSS.


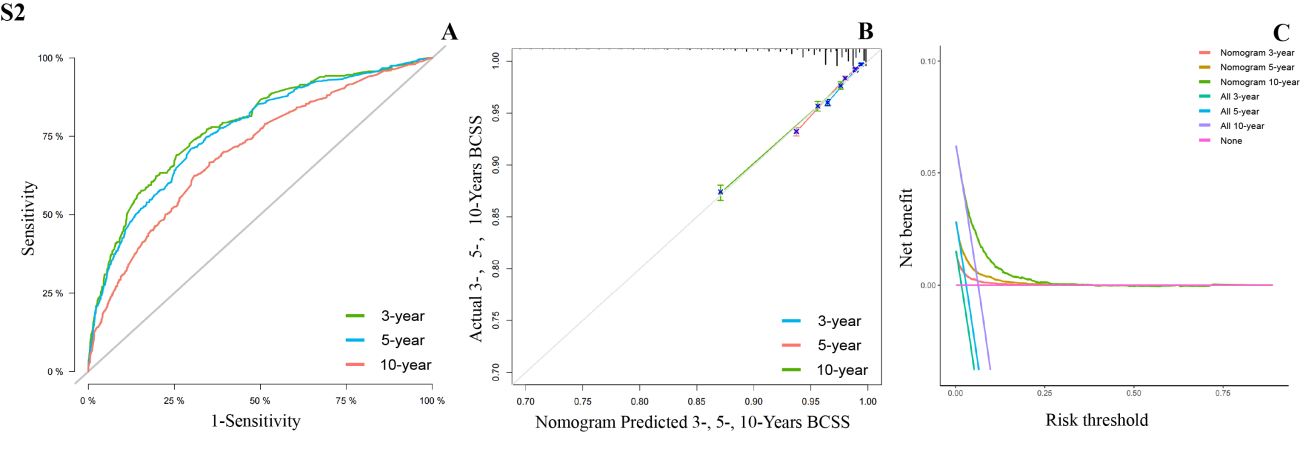


**Figure S3** **Survival curves comparing survival outcomes between the T1a group and the T1b group.** Kaplan-Meier curves were applied to compare survival between T1a and T1b groups. (A) The status of OS in the subgroups of T1. (B) The status of BCSS in the subgroups of T1.


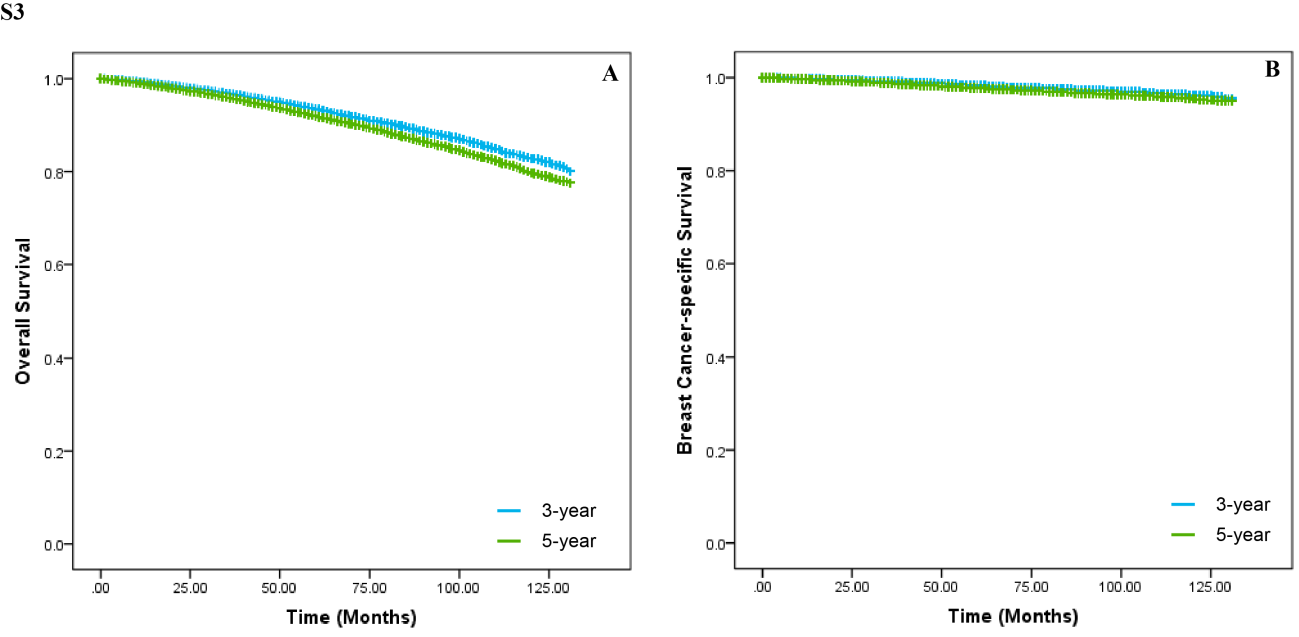

Supplement: Supplementary file 1 [file mmc1.docx]
